# T1 Stage Clear Cell Renal Cell Carcinoma: A CT-Based Radiomics Nomogram to Estimate the Risk of Recurrence and Metastasis

**DOI:** 10.3389/fonc.2020.579619

**Published:** 2020-11-04

**Authors:** Bing Kang, Cong Sun, Hui Gu, Shifeng Yang, Xianshun Yuan, Congshan Ji, Zhaoqin Huang, Xinxin Yu, Shaofeng Duan, Ximing Wang

**Affiliations:** ^1^ School of Medicine, Shandong University, Jinan, China; ^2^ Department of Radiology, Shandong Provincial Hospital Affiliated to Shandong University, Jinan, China; ^3^ Department of Radiology, Shandong Medical Imaging Research Institute, Jinan, China; ^4^ GE Healthcare, Shanghai, China

**Keywords:** clear cell renal cell carcinoma, recurrence, neoplasm metastasis, computed tomography, prediction model****

## Abstract

**Objectives:**

To develop and validate a radiomics nomogram to improve prediction of recurrence and metastasis risk in T1 stage clear cell renal cell carcinoma (ccRCC).

**Methods:**

This retrospective study recruited 168 consecutive patients (mean age, 53.9 years; range, 28–76 years; 43 women) with T1 ccRCC between January 2012 and June 2019, including 50 aggressive ccRCC based on synchronous metastasis or recurrence after surgery. The patients were divided into two cohorts (training and validation) at a 7:3 ratio. Radiomics features were extracted from contrast enhanced CT images. A radiomics signature was developed based on reproducible features by means of the least absolute shrinkage and selection operator method. Demographics, laboratory variables (including sex, age, Fuhrman grade, hemoglobin, platelet, neutrophils, albumin, and calcium) and CT findings were combined to develop clinical factors model. Integrating radiomics signature and independent clinical factors, a radiomics nomogram was developed. Nomogram performance was determined by calibration, discrimination, and clinical usefulness.

**Results:**

Ten features were used to build radiomics signature, which yielded an area under the curve (AUC) of 0.86 in the training cohort and 0.85 in the validation cohort. By incorporating the sex, maximum diameter, neutrophil count, albumin count, and radiomics score, a radiomics nomogram was developed. Radiomics nomogram (AUC: training, 0.91; validation, 0.92) had higher performance than clinical factors model (AUC: training, 0.86; validation, 0.90) or radiomics signature as a means of identifying patients at high risk for recurrence and metastasis. The radiomics nomogram had higher sensitivity than clinical factors mode (McNemar’s chi-squared = 4.1667, p = 0.04) and a little lower specificity than clinical factors model (McNemar’s chi-squared = 3.2, p = 0.07). The nomogram showed good calibration. Decision curve analysis demonstrated the superiority of the nomogram compared with the clinical factors model in terms of clinical usefulness.

**Conclusion:**

The CT-based radiomics nomogram could help in predicting recurrence and metastasis risk in T1 ccRCC, which might provide assistance for clinicians in tailoring precise therapy.

## Introduction

Clear cell renal cell carcinoma (ccRCC) is the most common subtype of kidney cancer, whose incidence has been continuously increasing over the last few decades (1, 2). This trend is largely attributed to the widespread use of advantage radiologic diagnostic techniques (CT and ultrasound), as well as the popularization in regular checkups, allowing that most ccRCCs could be detected at T1 stage. ccRCC patients are at high risk of metastasis and recurrence (3). The incidence of RCC recurrence following nephrectomy has been reported to be 7% with a median time of 38 months for T1 tumors, 26% with a median time of 32 months for T2 disease, and 39% with a median time to recurrence at 17 months for T3 tumors (4). Tumor-node-metastasis stage and pathological grade are generally adopted to estimate the risk of tumor recurrence in patients with ccRCC after surgical operation. Nevertheless, distinct outcomes are demonstrated in patients with equivalent tumor-node-metastasis stage and pathological grade (5–8).

According to the European Association of Urology guidelines (1), localized T1 stage tumors are best managed by partial nephrectomy. At the same time, active surveillance can be offered to those patients of older age with co-morbidities, harboring a single kidney and/or those who are unwilling to undergo a major surgical operation. However, the tumor biology of T1 stage ccRCC keeps poorly understood. It is reported that a subset of patients with more aggressive ccRCC may benefit from adjuvant targeted therapy according to a recent study (9). Therefore, the development of an accurate system to ascertain which patients are at truly higher risk of metastasis or recurrence is needed to allow for better patient selection of those who are most likely to benefit from adjuvant therapy. Several studies have noted that nomograms comprising merely clinical factors were applied to assess the prognosis of ccRCC after surgery (10, 11). However, some of the parameters used in the nomogram such as tumor necrosis and clinical presentation are subject to inter-observer variability. Hence, further research and validation are needed.

Radiomics is a promising technique using computerized quantitative imaging analysis to extract an enormous quantity of image-related features, such as intensity, geometry, and texture, from medical images (12, 13). It has been increasingly reported that radiomics can be used for differentiating benign and malignant renal tumors, as well as discriminating high and low Fuhrman nuclear ccRCC (14–18). However, to the best of our knowledge, no study has evaluated radiomics for its ability to predict the aggressive potential of ccRCC.

The purpose of this study is to develop and validate a radiomics nomogram that incorporates the radiomics signature and the clinical factors to improve preoperative prediction of recurrence and metastasis risk in T1 stage ccRCC.

## Materials and Methods

### Institutional Board Approval

The institutional review board of our hospital approved this single-center retrospective study. The requirement for obtaining informed consent was waived.

### Patients

**Figure 1:**
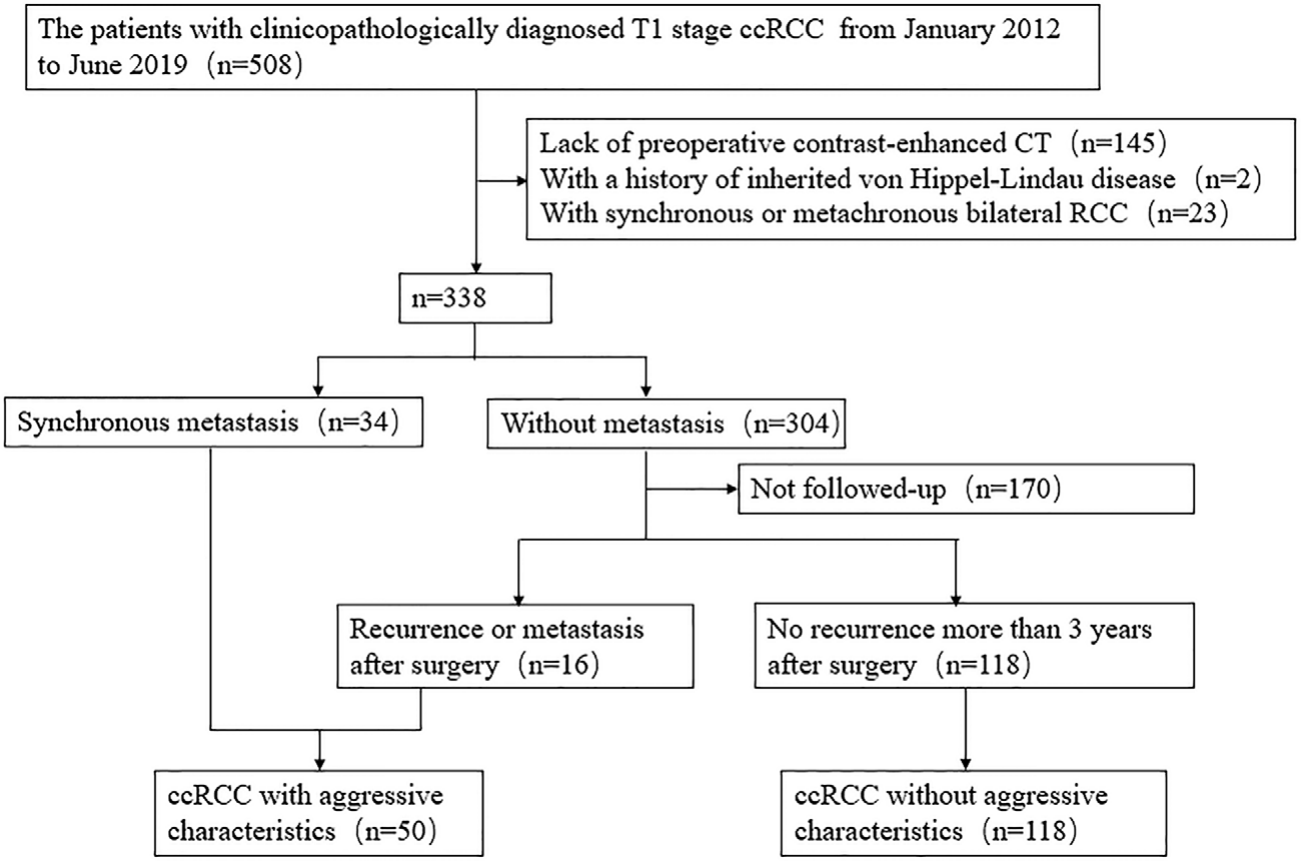
Recruitment pathway for patients in this study. CcRCC, clear cell renal cell carcinoma.

Data for surgically and pathologically confirmed ccRCC cases in our hospital were acquired from 1 January 2012 to 30 June 2019 by searching through our institutional database and medical record system. During the 7-year recruiting period, 508 consecutive patients with T1 stage ccRCC underwent surgical operation in our institution. A total of 145 patients were excluded due to absence of preoperative contrast enhanced CT images, and 25 patients were excluded due to history of von Hippel-Lindau disease or bilateral RCC. Aggressive tumors were defined as tumors exhibiting synchronous metastasis (n = 34), or recurrence after surgery (n = 16). The patient recruitment pathway is presented in **Figure 1** and **Supplementary Information 1.1**. The end points of our study were time until detection of metastasis or recurrence and time to last follow-up if the patient was alive. A total of 50 patients were defined as aggressive ccRCC (including nine T1a tumors and 41 T1b tumors), and 118 patients were non-aggressive ccRCC (including 65 T1a tumors and 53 T1b tumors). The metastatic locations were the lung (n = 12), bone (n = 21), liver (n = 2), retroperitoneum (n = 2), adrenal gland (n = 1), both the bone and lung (n = 6), both the lung and brain (n = 2), both the lung and adrenal gland (n = 2), both the bone and liver (n = 1), and simultaneously the bone, lung, and adrenal gland (n = 1). A total of eight cases were confirmed by biopsy and histopathology, and the other cases were diagnosed by radiologic features, that is, there was an increase in volume or number of suspected metastases during follow-up. The patients were divided into two cohorts (training and validation) according to the proportion of 7:3 using computer-generated random numbers.

### CT Image Acquisition and Radiologic Evaluation

The details of image acquisition parameters are shown in **Supplementary Information 1.2** and **Supplementary Table 1**. Among the aggressive tumors, 33 (66.0%) patients underwent CT scans using a 320-detector CT scanner (Aquilion ONE, TOSHIBA) and 17 (34.0%) underwent a 64-detector CT scanner (Discovery, GE Healthcare). Among the non-aggressive tumors, 90 (76.3%) patients underwent CT scans using Aquilion ONE, and 28 (23.7%) patients underwent Discovery. Each CT study was analyzed by a radiology resident (Reader 1, BK) and a radiologist (Reader 2, XW) with 5 and 20 years of experience in abdominal imaging, respectively. Aware of the diagnosis of ccRCC but blinded to the radiological reports and pathologic details, the two researchers construed the following CT features by consensus: the maximum diameter of tumor on the axial CT image; tumor location (exophytic or not, exophytic meaning >50% outside renal parenchyma); tumor polarity (superior or inferior or middle); and tumor side (left or right). The maximum diameter of the tumor was measured by the two radiologists, and the average value was applied to the evaluation. For those qualitative parameters (including tumor location, polarity and side), in the event of disagreement, the two readers jointly reviewed the findings to reach a consensus for further analysis.

### Development of Clinical Factor Model

Univariate logistic regression analysis was applied to the clinical factors, including clinical data (sex, age, and Fuhrman grade), laboratory variables (hemoglobin, platelet, neutrophils, albumin, and calcium), and CT features to find the factor that significantly affected the event occurrence probability (p < 0.05). Then a multiple logistic regression analysis with a step-wise backwards elimination was subsequently applied to build the clinical factors model. Odds ratios (ORs) as estimates of relative risk with 95% confidence intervals (CIs) were calculated for each risk factor.

### Segmentation of Tumor Images and Radiomics Feature Extraction

**Figure 2:**
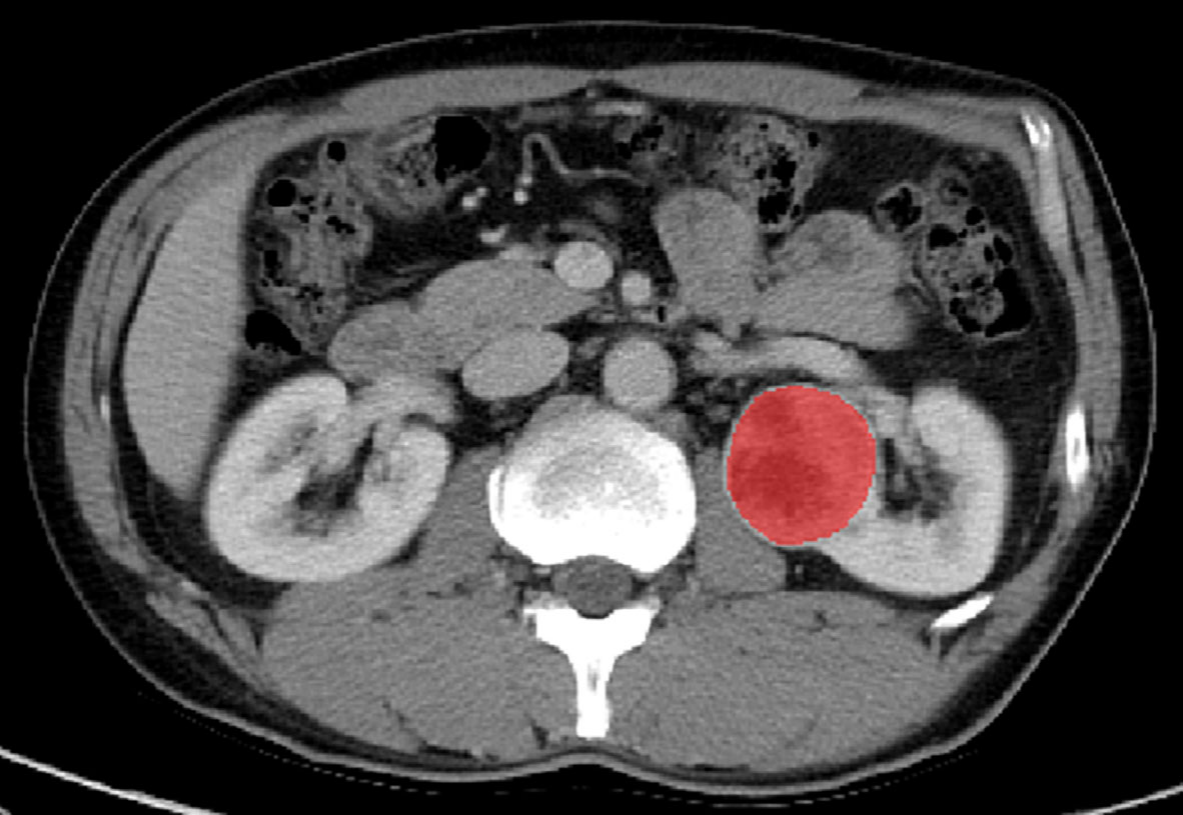
Manual segmentation of the tumor on the center axial slice of the clear cell renal cell carcinoma (ccRCC).

ITK-SNAP software (Version 3.6.0, www.itksnap.org) was used for segmentation of tumors. A defined polygonal region-of-interest was delineated on the center slice of the ccRCC on corticomedullary phase (CMP) and nephrographic phase (NP) images, avoiding covering the paratumoral renal parenchyma and perinephric fat (**Figure 2**).

AK software (AnalysisKit 3.2.0; GE Healthcare, China) was used to extract a total of 396 radiomics features from the region-of-interest for one phase. The radiomics features are detailed in **Supplementary Information 1.3**.

Inter- and intra-class correlation coefficients (ICCs) were calculated to estimate the inter-observer reliability and intra-observer reproducibility of features extraction. 20 cases of CT images containing six aggressive ccRCCs and 14 non-aggressive ccRCCs were randomly chosen; region-of-interest segmentation was drawn by one radiology resident (Reader 1, BK) and one radiologist (Reader 2, XW) independently; both were aware of the diagnosis of ccRCC but were blinded to the pathologic details. Reader 1 then repeated the contouring procedure 8 weeks after the initial analysis to assess the agreement of feature extraction. The remaining image segmentation was performed by Reader 1.

### Development of Radiomics Signature and Radiomics Nomogram

The prevention of the overfitting of the signature can be realized through the conduction of dimension reduction of the features before signature construction. Only were the radiomics chosen to be kept when meeting a criterion of inter- and intra-observer ICCs greater than 0.75, then the minimum-Redundancy Maximum-Relevancy method was performed to eliminate the redundant and irrelated features and kept 30 features. The remaining features were enrolled into the least absolute shrinkage and selection operator (LASSO) regression model to select the most valuable features in the training cohort. Then the radiomics signature (Radiomics score) was calculated by summing the selected feature values weighted by their corresponding features.

To provide a more individualized predictive model, a nomogram combining the final radiomics signature and significant clinical variables were built in the training cohort. The calibration of the nomogram was evaluated with a calibration curve. The Hosmer–Lemeshow test was conducted to assess the goodness-of-fit of the nomogram. A radiomics nomogram score for each patient was obtained in the training and validation cohorts.

### Assessment of the Performance of Different Models

The predictive accuracy of the clinical factors model, radiomics signature, and radiomics nomogram for differentiating aggressive ccRCC from non-aggressive ccRCC was quantified by the area under the receiver operating characteristics (ROC) curve (AUC) in both the training and validation sets. Decision curve analysis was used to calculate the net benefits for a range of threshold probabilities in the whole cohort to assess the clinical usefulness of the nomogram.

### Statistical Analysis

Statistical tests were performed using R statistical software (version 3.5.1, https://www.r-project.org). Univariate logistic regression analysis was applied to find the factor that significantly affected the event occurrence probability (p<0.05). Group differences are figured out by means of univariate analysis, which consists of chi-square test or Fisher exact test for categorical variables and Mann–Whitney U test for continuous variables, where appropriate. The LASSO-logistic regression model was used to select the features and construct the radiomics signature. A linear combination of the selected features and the product of the corresponding weighting coefficients was utilized to calculate the radiomics score of each patient. A multiple logistic regression analysis was applied to develop the radiomics nomogram by using the statistically significant clinical characteristics and the radiomics signature as input variables. ROC analysis was conducted to evaluate the performance of each model, and the differences in the AUC values between different models were estimated using the Delong’s test. Besides, McNemar test was used to compare the sensitivity and specificity between the clinical factors model and radiomics nomogram. The Hosmer–Lemeshow test and a decision curve were used to evaluate and validate the radiomics nomogram results. A two-tailed P < 0.05 was indicative of statistical significance.

## Results

### Clinical Factors of the Patients and Construction of the Clinical Factor Model

The patients’ demographic baseline characteristics (mean age, 53.9 years; age range, 28–76 years; 43 women) are summarized in **Table 1**. No differences were detected in clinical characteristics between the training and validation cohorts (p = 0.124–0.948). The rates of aggressive ccRCC were 29.7% (35 of 118) and 30% (15 of 50) in the training cohort and validation cohort, respectively, whereas no statistically significant difference was found between the two cohorts. The results of multiple logistic regression analysis are listed in **Table 2**, which suggested that only maximum diameter and albumin remained as independent predictors of aggressive ccRCC (p < 0.05). Tumors with larger maximum diameter (OR, 1.61; 95% CI, 1.14–2.28) or lower albumin (OR, 0.79; 95% CI, 0.69–0.92) were likely to be aggressive ccRCC. The clinical factors model was constructed using the backward step-wise multivariate logistic regression with Akaike information criterion (AIC) as criterion. This method only considered the AIC rather than the p value of each clinical factor so that the method determined the optimized feature subset. Finally, the sex, maximum diameter, neutrophils, and albumin were incorporated into the institution of the clinical factors model.

**Table 1:** Characteristics of patients in the training and validation cohorts.

Clinical factors	Training cohort (n = 118)			Validation cohort (n = 50)		
	Aggressive ccRCC	Non-aggressive ccRCC	p	Aggressive ccRCC	Non-aggressive ccRCC	p
Sex			0.092			0.843
Men	31 (88.6)	60 (72.3)		11 (73.3)	23 (65.7)	
Women	4 (11.4)	23 (27.7)		4 (26.7)	12 (34.3)	
Age (years)^*^	56.8 ± 9.6 (30–72)	53.0 ± 11.5 (28–76)	0.088	53.6 ± 11.1 (31–68)	53.4 ± 11.5 (30–76)	0.948
Nephrectomy type Partial Radical	10 (28.6) 25 (71.4)	37 (44.6) 46 (55.4)	0.105	5 (33.3) 10 (66.7)	19 (54.3) 16 (45.7)	0.174
Polarity			0.233			0.857
Superior	10 (28.6)	22 (26.5)		5 (33.3)	9 (25.7)	
Middle	11 (31.4)	39 (47.0)		6 (40.0)	16 (45.7)	
Inferior	14 (40.0)	22 (26.5)		4 (26.7)	10 (28.6)	
Location			0.076			0.090
Exophytic	23 (65.7)	38 (45.8)		13 (86.7)	20 (57.1)	
Not exophytic	12 (34.3)	45 (54.2)		2 (13.3)	15 (42.9)	
Side			1.000			1.000
Left	16 (45.7)	37 (44.6)		7 (46.7)	18 (51.4)	
Right	19 (54.3)	46 (55.4)		8 (53.3)	17 (48.6)	
Maximum diameter (cm)^*^	5.2 ± 1.5 (1.5–7.0)	4.0 ± 1.4 (1.2–7.0)	<0.001	5.3 ± 1.4 (2.9–7.0)	3.8 ± 1.3 (1.9–6.9)	<0.001
Fuhrman grade			0.059			NA
1	0 (0.0)	11 (13.3)		1 (6.7)	5 (14.3)	
2	31 (88.6)	64 (77.1)		11 (73.3)	26 (74.3)	
3	3 (8.6)	8 (9.6)		3 (20.0)	4 (11.4)	
4	1 (2.9)	0 (0.0)		0 (0.0)	0 (0.0)	
Hemoglobin (g/L)^*^	139.5 ± 21.5 (81–175)	143.1 ± 16.9 (71–169)	0.328	141.0 ± 19.5 (109–167)	149.1 ± 15.6 (117–178)	0.117
Platelet (10^9^/L)^*^	275.6 ± 80.2 (190–589)	236.0 ± 56.2 (115–423)	0.002	305.8 ± 140.9 (172–747)	234.4 ± 45.7 (154–399)	0.007
Neutrophils (10^9^/L)^*^	5.1 ± 2.5 (2.11–13.19)	3.7 ± 1.2 (0.68–9.01)	<0.001	5.1 ± 2.2 (1.73–10.07)	3.7 ± 1.0 (1.87–6.15)	0.002
Albumin (g/L)^*^	38.7 ± 5.3 (26.7–49.1)	42.9 ± 2.9 (34.8–49.5)	<0.001	39.9 ± 4.0 (33.8–46.4)	43.3 ± 3.2 (36.1–48.4)	0.001
Calcium (mmol/L)^*^	2.3 ± 0.3 (1.9–3.28)	2.3 ± 0.1 (1.98–2.78)	0.433	2.3 ± 0.1 (2.13–2.66)	2.3 ± 0.1 (2.1–2.54)	0.937
Median Rad-score^†^	−0.3 (−0.7, 0.5)	−1.7 (−2.2, −0.9)	<0.001	−0.5 (−0.8, 0.1)	−1.7 (−2.3, −1.1)	<0.001

Unless otherwise indicated, data are number of patients and data in parentheses are percentages. The sum of percentages may not be 100% because of rounding. ccRCC, clear cell renal cell carcinoma; NA, not available.

*Data are mean ± standard deviation; data in parentheses are range.

^†^Data in parentheses are interquartile range.

**Table 2:** Risk factors for aggressive ccRCC.

Variable	Clinical model		Radiomics nomogram	
	Odds ratio (95% CI)	P value	Odds ratio (95% CI)	P value
Sex	0.27 (0.05–1.36)	0.112	0.34 (0.05–2.25)	0.265
Maximum diameter	1.61 (1.14–2.28)	0.007	0.96 (0.62–1.49)	0.849
Neutrophils	1.41 (0.99–2.02)	0.058	1.44 (0.94–2.22)	0.093
Albumin	0.79 (0.69–0.92)	0.002	0.81 (0.69–0.95)	0.008
Rad-score	NA	NA	3.76 (1.77–7.99)	<0.001

Data are results of the multiple logistic regression analysis. ccRCC, clear cell renal cell carcinoma; NA, not available; CI, confidence interval.

### Feature Extraction, Selection, and Radiomics Signature Establishment

**Figure 3:**
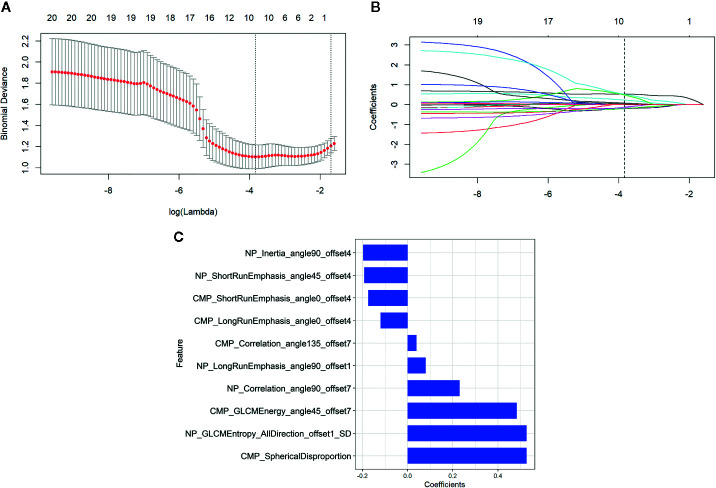
Radiomics feature selection by using the least absolute shrinkage and selection operator (LASSO) logistic regression. **(A)** Selection of the tuning parameter (*λ*) in the LASSO model. An optimal *λ* value of 0.022 (vertical dash line) with log(*λ*) = −3.836 was selected. **(B)** The feature coefficients varied according to log(*λ*). **(C)** The selected features with nonzero coefficients and their coefficients.

Consistent inter- and intra-observer agreement was found in 654 features (ICCs, 0.8279–0.9595) among the total of 792 radiomics features extracted from CMP and NP CT images. Thirty radiomics features exhibiting significant differences between aggressive ccRCC and non-aggressive ccRCC by minimum-Redundancy Maximum-Relevancy were enrolled into the LASSO logistic regression model to select the most valuable features (**Figures 3A, B**). Finally, the selected 10 radiomics features were displayed in **Figure 3C**. The radiomics score was attained with the following formula:

$$\begin{array}{l} “\text{Radiomics score}=0.527\times \text{NP\_GLCMentropy\_AllDirection\_offset}1\text{\_SD}+0.23\\ \times \text{NP\_Correlation\_angle}90\text{\_offset}7-0.174\times \text{CMP\_ShortRunEmphasis\_angle}0\text{\_offset}4-0.197\\ \times \text{NP\_Inertia\_angle}90\text{\_offset}4+0.483\times \text{CMP\_GLCMEnergy\_angle}45\text{\_offset}7+0.527\\ \times \text{CMP\_SphericalDisproportion}-0.119\times \text{CMP\_LongRunEmphasis\_angle}0\text{\_offset}4+0.08\\ \times \text{NP\_LongRunEmphasis\_angle}90\text{\_offset}1-0.192\times \text{NP\_ShortRunEmphasis\_angle}45\text{\_offset}4+0.04\\ \times \text{CMP\_Correlation\_angle}135\text{\_offset}7-\;1.066” \end{array}$$

**Figure 4:**
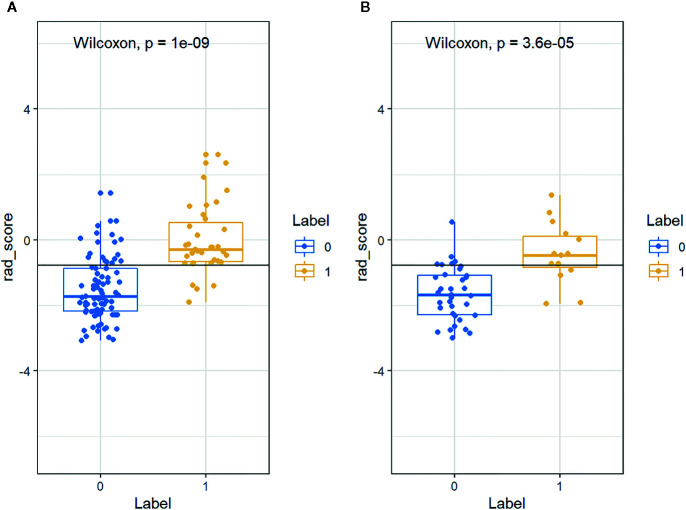
The distributions of the Rad-score for each patient in the **(A)** training and **(B)** validation cohorts. Blue and yellow represent non-aggressive clear cell renal cell carcinoma (ccRCC) and aggressive ccRCC, respectively.

**Figure 5:**
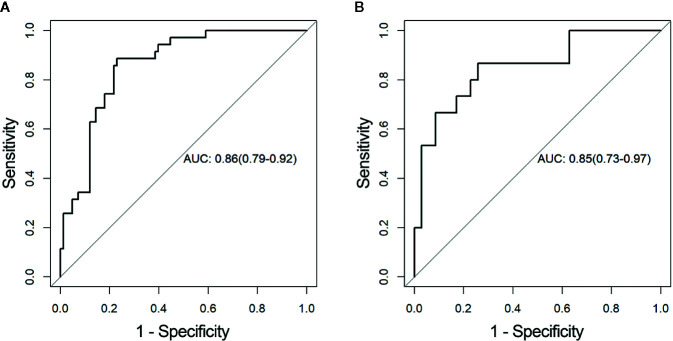
Receiver operating characteristic (ROC) curves of the radiomics signature in the **(A)** training and **(B)** validation cohorts, respectively. AUC, area under the receiver operating characteristic curve.

The distributions of the radiomics score for each patient in training and validation cohorts are displayed in **Figure 4**. Radiomics score [median (interquartile range)] differed significantly between the aggressive and non-aggressive ccRCC groups in the training cohort [−0.3 (−0.7, 0.5) *vs*. −1.7 (−2.2, −0.9), respectively, p < 0.001]; this finding was verified in the validation cohort [−0.5 (−0.8, 0.1) *vs*. −1.7 (−2.3, −1.1), respectively, p < 0.001]. ROC curves of radiomics signature are displayed in **Figure 5**. The radiomics signature yielded an AUC of 0.86 (95% CI: 0.79, 0.92) in the training cohort and 0.85 (95%CI: 0.73, 0.97) in the validation cohort, showing favorable predictive efficacy. Furthermore, we applied leave group out cross validation (LGOCV) to validate the model’s robustness. The mean AUC, accuracy, sensitivity, specificity of LGOCV were 0.74, 0.72, 0.79, 0.69, respectively.

### The Radiomics Nomogram Establishment and Assessment of the Performance of Different Models

**Figure 6:**
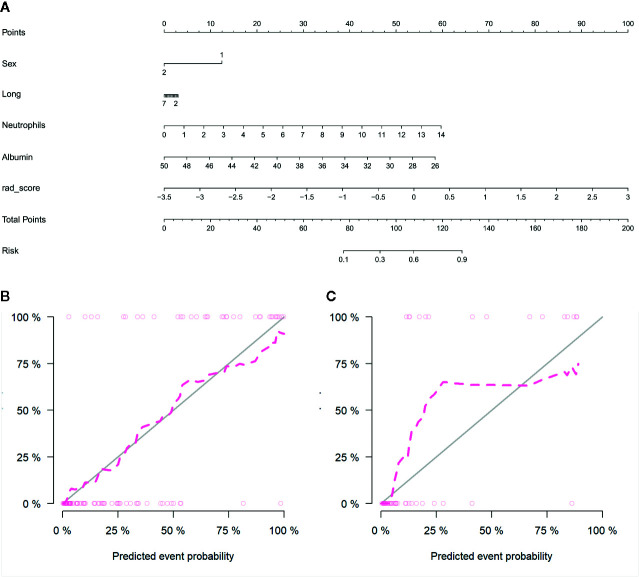
Radiomics nomogram developed with receiver operating characteristic (ROC) curves and calibration curves. **(A)** The radiomics nomogram, combining sex, tumor maximum diameter, neutrophils, albumin, and Rad-score, developed in the training set. The nomogram calibration curves in the training **(B)** and validation **(C)** sets. Calibration curves indicate the goodness-of-fit of the model. The closer the pink line approaches the gray line, the better agreement between the predictive probabilities and the observed probabilities.

By incorporating the sex, maximum diameter, neutrophil count, albumin count, and radiomics score, a radiomics nomogram was developed in the training cohort (**Figure 6A**). The calibration curve of the radiomics nomogram demonstrated good agreement between the predicted and expected probabilities for aggressive ccRCC in training cohort (**Figure 6B**). The p values of Hosmer–Lemeshow test were 0.45 and 0.11 in training and validation cohorts respectively.

**Figure 7:**
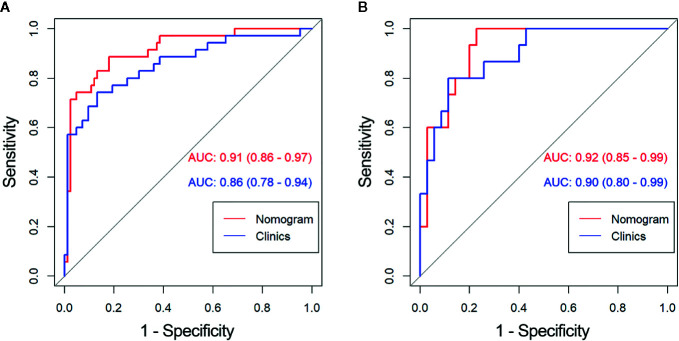
Comparison of receiver operating characteristic (ROC) curves between the radiomics nomogram and clinical model for the prediction of aggressive clear cell renal cell carcinoma (ccRCC) in the **(A)** training and **(B)** validation cohorts. AUC, area under the receiver operating characteristic curve.

The diagnostic performance of every model is demonstrated in **Table 3**. The ROC curves of radiomics nomogram and clinical factors model are exhibited in **Figure 7**. In the training cohort, the radiomics nomogram showed the highest discrimination, with an AUC of 0.91 (95% CI: 0.86, 0.97); the observed AUC value was slightly higher than that of the clinical factors model [AUC, 0.86 (95% CI: 0.78, 0.94); p = 0.051]. In the validation cohort, the radiomics nomogram [AUC, 0.92 (95% CI: 0.85, 0.99)] also achieved more satisfactory predictive efficacy than the clinical factors model [AUC, 0.90 (95% CI: 0.80, 0.99)], although the difference was not statistically significant (p = 0.401). We then used McNemar test for comparison of the sensitivity and specificity between the clinical factors model and radiomics nomogram and found that the radiomics nomogram had higher sensitivity than the clinical factors model (100.0 *vs*. 60.0%, McNemar’s chi-squared = 4.1667, p = 0.04). However, the radiomics nomogram had a little lower specificity than the clinical factors model, whereas the difference was not statistically significant (77.1 *vs*. 91.4%, McNemar’s chi-squared = 3.2, p = 0.07). The nomogram score was acquired using the following formula:

$$\begin{array}{l} “\text{Nomogram score}=8.6938-1.0699\;\times \;\text{Sex}-0.0430\;\;\times \text{Maximum diameter}+0.3671\times \text{Neutrophils}-0.2096\\ \times \;\text{Albumin}\;+\;1.3243\;\times \;\text{Radiomics score}” \end{array}$$

**Table 3:** Results of radiomics nomogram, radiomics signature, and the clinical model predictive ability for distinguishing between aggressive ccRCC and non-aggressive ccRCC.

Variables		AUC(95% CI)	Sensitivity*	Specificity*	Accuracy*
Clinical model	Training cohort	0.86(0.78–0.94)	74.3(26/35)	86.7(72/83)	83.1(98/118)
	Validation cohort	0.90(0.80–0.99)	60.0(9/15)	91.4(32/35)	82.0(41/50)
Radiomics signature	Training cohort	0.86(0.79–0.92)	88.6(31/35)	77.1(64/83)	80.5(95/118)
	Validation cohort	0.85(0.73–0.97)	73.3(11/15)	82.9(29/35)	80.0(40/50)
Radiomics nomogram	Training cohort	0.91(0.86–0.97)	88.6(31/35)	81.9(68/83)	83.9(99/118)
	Validation cohort	0.92(0.85–0.99)	100.0(15/15)	77.1(27/35)	84.0(42/50)

CI, confidence interval.

*Numbers in parentheses were used to calculate percentages.

**Figure 8:**
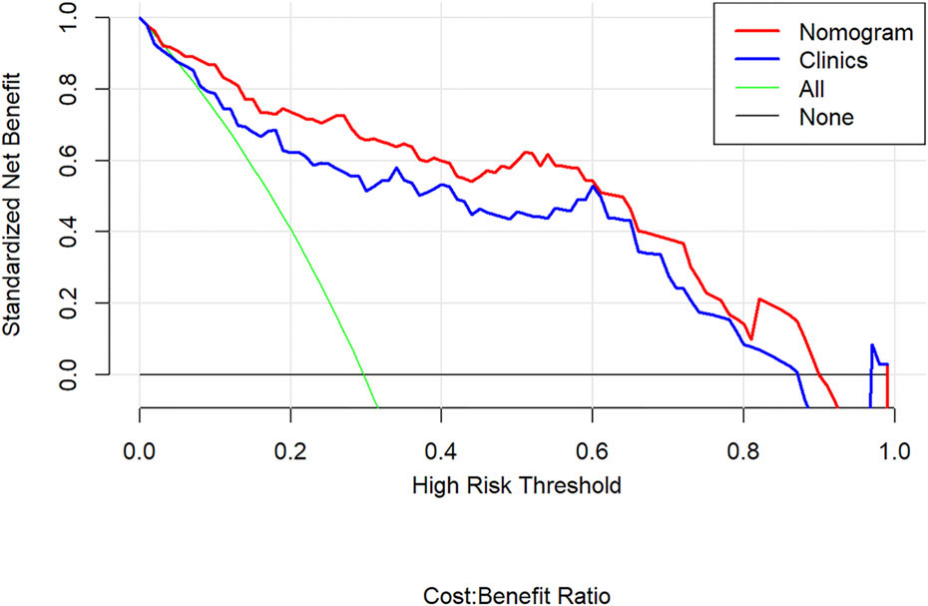
Decision curve analysis for the radiomics nomogram. The y-axis shows the net benefit; x-axis shows the threshold probability. The red line and blue line represent the net benefit of the radiomics nomogram and the clinical factor model, respectively. The green line indicates the hypothesis that all patients had aggressive clear cell renal cell carcinoma (ccRCC). The black line represents the hypothesis that no patients had aggressive ccRCC. The decision curves indicate that the application of radiomics nomogram to predict aggressive ccRCC adds more benefit than treating all or none of the patients, and clinical factor model, across the full range of reasonable threshold probabilities.

The decision curve analyses for the clinical factor model and radiomics nomogram are presented in **Figure 8**. It showed that the radiomics nomogram had a higher overall net benefit in differentiating aggressive ccRCC from non-aggressive ccRCC than the clinical factor model across the full range of reasonable threshold probabilities.

## Discussion

In this retrospective analysis, we developed a radiomics nomogram that incorporates four clinical factors and radiomics signature for noninvasive, individualized prediction of recurrence and metastasis risk in patients with clinical T1 stage ccRCC, which can enable physicians to select reasonable treatment tactics and individualized monitoring protocols to improve clinical outcomes. To the best of our knowledge, this is the first prediction model developed to predict recurrence and metastasis risk in T1 stage ccRCC using CT-based radiomics. The proposed radiomics nomogram demonstrated favorable discrimination in both the training cohort (AUC, 0.91) and the validation cohort (AUC, 0.92), indicating that it has better predictive performance than the clinical factor model (AUC: training, 0.86; validation, 0.90) or the radiomics signature (AUC: training, 0.86; validation, 0.85). The radiomics nomogram had higher sensitivity than the clinical factors model (100.0 *vs.* 60.0%, McNemar’s chi-squared = 4.1667, p = 0.04) and a little lower specificity than the clinical factors model (77.1 *vs*. 91.4%, McNemar’s chi-squared = 3.2, p = 0.07).

Most patients with T1 stage ccRCC will have excellent outcomes following resection or active surveillance, with a 97% 5-year survival imaging. Nevertheless, evaluating the recurrence and metastasis risk of ccRCC only by tumor stage is insufficient because some T1 ccRCC can be lethal once the tumor exhibits synchronous metastasis or recurrence (19, 20). Actually, the incidence of T1 RCC recurrence after nephrectomy has been reported to be 7% with a median time of 38 months (4). Wei et al. (21) developed a classifier based on single-nucleotide polymorphisms to predict recurrence risk in RCC and showed that “recurrence risk of the subgroup of the classifier-defined high risk in stage I or II was higher than the classifier-defined low risk in stage III”. Currently, management of T1 ccRCC depends on the surgeon’s discretion based on clinical and pathological parameters related to aggressive potential of the tumor (11, 22, 23). Prognostic factors and predictive models for RCC patients’ outcomes have been reported previously by multiple investigators (24–26). Park et al. (27) reviewed preoperative laboratory data in 747 RCC patients and revealed that clinical information supporting aggressive ccRCC included an older age, larger size, lower hemoglobin, albumin, and calcium, as well as higher platelet and neutrophil. However, few radiologic parameters have been reported as prognostic factors of ccRCC in contrast to pathological markers. We enrolled these variables in this study, and found maximum diameter, neutrophil, and albumin were significantly different between the two groups, which was consistent with previous studies. However, affected by the radiomics score, the maximum diameter in our nomogram was less important. Besides, we extracted the radiomics features also containing the geometry features which features also reflected the maximum diameter but after filtering the features, we found the maximum tumor diameter correlated features were abandoned, which meant the remaining features had more value in our paper. Compared with the clinical factors model that only relied on clinical data and CT features, the final radiomics nomogram model achieved higher prediction performance for aggressive ccRCC. The decision curve analysis revealed that using the radiomics nomogram to differentiate aggressive ccRCC from non-aggressive ccRCC presents more notable benefits than solely relying on clinical factor model.

A prognostic multigene signature (28) has been developed to predict recurrence risk in ccRCC, identifying that aggressive ccRCC are characterized by reduced angiogenic dependence. The present paradigm of ccRCC imaging interpretation relies on a visual process, which comprises evaluation of the shape, margin, as well as degree and heterogeneity of enhancement. Junki et al. (29) enrolled 88 patients with T1 stage ccRCC, including seven patients that had recurrence after nephrectomy, and revealed that tumor enhancement in the NP of CT was a predictive factor for recurrence. However, these subjective approaches do not adequately reflect discrepancies in the angiogenesis (30). To the best of our knowledge, the medical images are the product of procedures appearing at the level of the gene and molecule. As such, imaging parameters acquired from advanced image procedure and analysis, such as radiomics, can address the underlying molecular and genotypic basis of the tissue (31–33). Recently, radiomics have been reported for distinguishing benign and malignant renal tumors, predicting ccRCC Fuhrman grade and therapeutic response (32–38); radiomics related to the recurrence and metastasis risk in ccRCC have rarely been reported. A radiomics signature in our study was constructed using ten selected features including gray-level co-occurrence matrix (GLCM), run length matrix (RLM), and form factor matrix. Among the selected radiomics features, Spherical Disproportion, GLCMEntropy, and GLCMEnergy were the most significant and robust features associated with aggressive ccRCC. The Spherical Disproportion feature quantified the degree of irregularity in the tumor boundary. An irregular tumor boundary could be a sign of poor survival (39). Entropy is a parameter describing the complexity of an image, which means the larger entropy value is indictive of a more complex tumor (40). Compared with the subjective CT findings, our radiomics nomogram based on the quantitative analysis of image features shows greater predictive power.

Our study has filled a gap in the literature on recurrence and metastasis risk of T1 ccRCC in the setting of radiomics. Unlike previous work, our radiomics nomogram could provide beneficial information for preoperative prediction of T1 stage aggressive ccRCC to estimate the necessity of adjuvant therapy. Our study may have important clinical significance because the risk of recurrence and metastasis is one of the most meaningful prognostic ingredients, which is associated with cancer-related overall survival after surgical operation (22). Three large clinical trials (9, 41, 42) evaluated the use of adjuvant tyrosine kinase inhibitors in ccRCC, concluding that patient selection is one of important factors to maximize the benefit of adjuvant therapy, which means it is critical to choose a population at high risk of cancer recurrence. The patients with non-aggressive ccRCC could be cured by surgery alone, and adjuvant therapy is of no necessity and is not additionally beneficial; while those patients with aggressive RCC, who were at high risk for tumor recurrence, would have a longer duration of disease-free survival if they were receiving adjuvant treatment. Therefore, accurate evaluation of the recurrence risk cannot only assist in patient consultation and manage treatment but also help guide follow-up and diminish overtreatment in low-risk patients. Our radiomics nomogram would allow for stratifying patients diagnosed with T1 stage ccRCC for their follow-up schedule. For patients with aggressive ccRCC, more frequent monitoring in postoperative follow-up is of significant necessity.

There are several limitations to our study. First, owing to the limitation of the retrospective study and small number of cases, the follow-up time we used was at least 3 years. Although recurrence of ccRCC after surgery occurs within 3 years in most patients, there still some patients developed recurrence >3 years after surgery. It would be more interesting to enroll patients without recurrence evidence for more than 5 or 10 years and further prospective research would focus on these cases. Second, as a single-center study, the patient population was relatively homogeneous and small. During the 7-year recruiting period, 168 T1 stage ccRCC were eligible for our study, including 74 T1a tumors and 94 T1b tumors. There is not enough data to differentiate T1a and T1b tumors to perform a stratified analysis, which is paramount. A large-scale independent prospective multicenter study is needed to evaluate the generalizability of the results, as well as take into account the differentiation between the T1a tumors and T1b tumors. Third, only the largest two-dimensional region-of-interest was applied for our study. Although it is reported that three-dimensional radiomics analysis appeared more indicative of tumor heterogeneity, we think that it would not be clinically practical owing to extra segmentation duration. Fourth, all of the images in this retrospective study underwent a fixed procedure instead of individualized optimal scan protocol, which may influence the image quality. The next step is to conduct prospective and standardized research. Optimal scanning time by using bolus tracking and individualized amount of contrast medium will be considered in our future study. Last, we defined aggressive ccRCC as tumor exhibiting synchronous metastasis or recurrence after surgery. However, there may be significant radiomical differences between patients with synchronous metastasis and recurrence. Furthermore, our prospective research on the radiomics nomogram for predicting recurrence risk after surgical operation is ongoing.

In conclusion, our study presented a CT-based radiomics nomogram that showed satisfactory performance in predicting recurrence and metastasis risk among patients diagnosed with T1 stage ccRCC, which can enable physicians to make more informed treatment decisions about adjuvant therapy. Radiomics nomogram, as a non-invasive and quantitative method, may serve as an efficient tool to complement the conventional procedures for clinical decision-making process.

## Data Availability Statement

The raw data supporting the conclusions of this article will be made available by the authors, without undue reservation.

## Ethics Statement

The studies involving human participants were reviewed and approved by Shandong Provincial Hospital. Written informed consent for participation was not required for this study in accordance with the national legislation and the institutional requirements.

## Author Contributions

Study conception and design: BK, CS, and XMW. Literature research: BK and HG. Data acquisition: BK, SY, XYuan, CJ, ZH, and XYu. Assessment of image feature: BK and XMW. Statistical analysis: BK and SD. Drafting the article: BK. Manuscript editing: BK, CS, and XMW. All authors contributed to the article and approved the submitted version.

## Funding

The present study was supported by a grant from the Taishan Scholars Project (XMW) and National Natural Science Foundation of China Grant (81871354, 81571672, and 81371548).

## Conflict of Interest

SD was employed by the company GE Healthcare.

The remaining authors declare that the research was conducted in the absence of any commercial or financial relationships that could be construed as a potential conflict of interest.

## References

[1] Ljungberg B Albiges L Abu-Ghanem Y Bensalah K Dabestani S Fernandez-Pello S European Association of Urology Guidelines on Renal Cell Carcinoma: The 2019 Update. Eur Urol (2019) 75:799–810. 10.1016/j.eururo.2019.02.011 30803729

[2] Znaor A Lortet-Tieulent J Laversanne M Jemal A Bray F International variations and trends in renal cell carcinoma incidence and mortality. Eur Urol (2015) 67:519–30. 10.1016/j.eururo.2014.10.002 25449206

[3] Brufau BP Cerqueda CS Villalba LB Izquierdo RS Gonzalez BM Molina CN Metastatic renal cell carcinoma: radiologic findings and assessment of response to targeted antiangiogenic therapy by using multidetector CT. Radiographics (2013) 33:1691–716. 10.1148/rg.336125110 24108558

[4] Levy DA Slaton JW Swanson DA Dinney CPN Stage specific guidelines for surveillance after radical nephrectomy for local renal cell carcinoma. J Urol (1998) 159:1163–7. 10.1097/00005392-199804000-00014 9507823

[5] Ha YS Park YH Kang SH Hong SH Hwang TK Byun SS Predictive factors for late recurrence in patients with stage T1 clear cell renal cell carcinoma: a multiinstitutional study. Clin Genitourin Cancer (2013) 11:51–5. 10.1016/j.clgc.2012.08.008 23017336

[6] Eggener SE Yossepowitch O Pettus JA Snyder ME Motzer RJ Russo P Renal cell carcinoma recurrence after nephrectomy for localized disease: predicting survival from time of recurrence. J Clin Oncol (2006) 24:3101–6. 10.1200/JCO.2005.04.8280 16809736

[7] Rini BI Campbell SC Escudier B Renal cell carcinoma. Lancet (2009) 373:1119–32. 10.1016/S0140-6736(09)60229-4 19269025

[8] Park JS Pierorazio PM Lee JH Lee HJ Lim YS Jang WS Gene Expression Analysis of Aggressive Clinical T1 Stage Clear Cell Renal Cell Carcinoma for Identifying Potential Diagnostic and Prognostic Biomarkers. Cancers (Basel) (2020) 12:222. 10.3390/cancers12010222 PMC7017065 31963294

[9] Ravaud A Motzer RJ Pandha HS George DJ Pantuck AJ Patel A Adjuvant Sunitinib in High-Risk Renal-Cell Carcinoma after Nephrectomy. N Engl J Med (2016) 375:2246–54. 10.1056/NEJMoa1611406 27718781

[10] Leibovich BC Blute ML Cheville JC Lohse CM Frank I Kwon ED Prediction of progression after radical nephrectomy for patients with clear cell renal cell carcinoma: a stratification tool for prospective clinical trials. Cancer (2003) 97:1663–71. 10.1002/cncr.11234 12655523

[11] Sorbellini M Kattan MW Snyder ME Reuter V Motzer R Goetzl M A postoperative prognostic nomogram predicting recurrence for patients with conventional clear cell renal cell carcinoma. J Urol (2005) 173:48–51. 10.1097/01.ju.0000148261.19532.2c 15592023

[12] Gillies RJ Kinahan PE Hricak H Radiomics: Images Are More than Pictures, They Are Data. Radiology (2016) 278:563–77. 10.1148/radiol.2015151169 PMC4734157 26579733

[13] Lambin P Leijenaar RTH Deist TM Peerlings J de Jong EEC van Timmeren J Radiomics: the bridge between medical imaging and personalized medicine. Nat Rev Clin Oncol (2017) 14:749–62. 10.1038/nrclinonc.2017.141 28975929

[14] Bektas CT Kocak B Yardimci AH Turkcanoglu MH Yucetas U Koca SB Clear Cell Renal Cell Carcinoma: Machine Learning-Based Quantitative Computed Tomography Texture Analysis for Prediction of Fuhrman Nuclear Grade. Eur Radiol (2019) 29:1153–63. 10.1007/s00330-018-5698-2 30167812

[15] Feng Z Rong P Cao P Zhou Q Zhu W Yan Z Machine learning-based quantitative texture analysis of CT images of small renal masses: Differentiation of angiomyolipoma without visible fat from renal cell carcinoma. Eur Radiol (2018) 28:1625–33. 10.1007/s00330-017-5118-z 29134348

[16] Hodgdon T McInnes MD Schieda N Flood TA Lamb L Thornhill RE Can Quantitative CT Texture Analysis be Used to Differentiate Fat-poor Renal Angiomyolipoma from Renal Cell Carcinoma on Unenhanced CT Images? Radiology (2015) 276:787–96. 10.1148/radiol.2015142215 25906183

[17] Lubner MG Stabo N Abel EJ Del Rio AM Pickhardt PJ CT Textural Analysis of Large Primary Renal Cell Carcinomas: Pretreatment Tumor Heterogeneity Correlates With Histologic Findings and Clinical Outcomes. AJR Am J Roentgenol (2016) 207:96–105. 10.2214/AJR.15.15451 27145377

[18] Sasiwimonphan K Takahashi N Leibovich BC Carter RE Atwell TD Kawashima A Small (<4 cm) renal mass: differentiation of angiomyolipoma without visible fat from renal cell carcinoma utilizing MR imaging. Radiology (2012) 263:160–8. 10.1148/radiol.12111205 22344404

[19] Hancock SB Georgiades CS Kidney Cancer. Cancer J (2016) 22:387–92. 10.1097/PPO.0000000000000225 27870681

[20] Campbell SC Novick AC Belldegrun A Blute ML Chow GK Derweesh IH Guideline for management of the clinical T1 renal mass. J Urol (2009) 182:1271–9. 10.1016/j.juro.2009.07.004 19683266

[21] Wei JH Feng ZH Cao Y Zhao HW Chen ZH Liao B Predictive value of single-nucleotide polymorphism signature for recurrence in localised renal cell carcinoma: a retrospective analysis and multicentre validation study. Lancet Oncol (2019) 20:591–600. 10.1016/S1470-2045(18)30932-X 30880070

[22] Adamy A Chong KT Chade D Costaras J Russo G Kaag MG Clinical characteristics and outcomes of patients with recurrence 5 years after nephrectomy for localized renal cell carcinoma. J Urol (2011) 185:433–8. 10.1016/j.juro.2010.09.100 21167521

[23] Park JS Lee HJ Cho NH Kim J Jang WS Heo JE Risk Prediction Tool for Aggressive Tumors in Clinical T1 Stage Clear Cell Renal Cell Carcinoma Using Molecular Biomarkers. Comput Struct Biotechnol J (2019) 17:371–7. 10.1016/j.csbj.2019.03.005 PMC6434066 30962867

[24] Frank I Blute ML Cheville JC Lohse CM Weaver AL Zincke H An outcome prediction model for patients with clear cell renal cell carcinoma treated with radical nephrectomy based on tumor stage, size, grade and necrosis: the SSIGN score. J Urol (2002) 168:2395–400. 10.1097/01.ju.0000035885.91935.d5 12441925

[25] Motzer RJ Mazumdar M Bacik J Berg W Amsterdam A Ferrara J Survival and prognostic stratification of 670 patients with advanced renal cell carcinoma. J Clin Oncol (1999) 17:2530–40. 10.1200/JCO.1999.17.8.2530 10561319

[26] Zisman A Pantuck AJ Dorey F Chao DH Gitlitz BJ Moldawer N Mathematical model to predict individual survival for patients with renal cell carcinoma. J Clin Oncol (2002) 20:1368–74. 10.1200/JCO.2002.20.5.1368 11870181

[27] Park YH Baik KD Lee YJ Ku JH Kim HH Kwak C Late recurrence of renal cell carcinoma >5 years after surgery: clinicopathological characteristics and prognosis. BJU Int (2012) 110:E553–8. 10.1111/j.1464-410X.2012.11246.x 22578274

[28] Rini B Goddard A Knezevic D Maddala T Zhou M Aydin H A 16-gene assay to predict recurrence after surgery in localised renal cell carcinoma: development and validation studies. Lancet Oncol (2015) 16:676–85. 10.1016/S1470-2045(15)70167-1 25979595

[29] Maehara J Nishie A Asayama Y Ishigami K Ushijima Y Takayama Y Tumor Enhancement on Dynamic CT: A Predictive Factor for Recurrence After Nephrectomy in Localized T1 Clear Cell Renal Cell Carcinoma. Anticancer Res (2018) 38:2377–83. 10.21873/anticanres.12486 29599364

[30] Deng Y Soule E Samuel A Shah S Cui E Asare-Sawiri M CT texture analysis in the differentiation of major renal cell carcinoma subtypes and correlation with Fuhrman grade. Eur Radiol (2019) 29:6922–9. 10.1007/s00330-019-06260-2 31127316

[31] Banerjee S Wang DS Kim HJ Sirlin CB Chan MG Korn RL A computed tomography radiogenomic biomarker predicts microvascular invasion and clinical outcomes in hepatocellular carcinoma. Hepatology (2015) 62:792–800. 10.1002/hep.27877 25930992 PMC4654334

[32] Pinker K Chin J Melsaether AN Morris EA Moy L Precision Medicine and Radiogenomics in Breast Cancer: New Approaches toward Diagnosis and Treatment. Radiology (2018) 287:732–47. 10.1148/radiol.2018172171 29782246

[33] Shinagare AB Krajewski KM Braschi-Amirfarzan M Ramaiya NH Advanced Renal Cell Carcinoma: Role of the Radiologist in the Era of Precision Medicine. Radiology (2017) 284:333–51. 10.1148/radiol.2017160343 28723287

[34] Chen T Ning Z Xu L Feng X Han S Roth HR Radiomics nomogram for predicting the malignant potential of gastrointestinal stromal tumours preoperatively. Eur Radiol (2019) 29:1074–82. 10.1007/s00330-018-5629-2 30116959

[35] Goh V Ganeshan B Nathan P Juttla JK Vinayan A Miles KA Assessment of response to tyrosine kinase inhibitors in metastatic renal cell cancer: CT texture as a predictive biomarker. Radiology (2011) 261:165–71. 10.1148/radiol.11110264 21813743

[36] Nie P Yang G Wang Z Yan L Miao W Hao D A CT-based radiomics nomogram for differentiation of renal angiomyolipoma without visible fat from homogeneous clear cell renal cell carcinoma. Eur Radiol (2020) 30:1274–84. 10.1007/s00330-019-06427-x 31506816

[37] Schieda N Lim RS Krishna S McInnes MDF Flood TA Thornhill RE Diagnostic Accuracy of Unenhanced CT Analysis to Differentiate Low-Grade From High-Grade Chromophobe Renal Cell Carcinoma. AJR Am J Roentgenol (2018) 210:1079–87. 10.2214/AJR.17.18874 29547054

[38] Zhang GM Shi B Xue HD Ganeshan B Sun H Jin ZY Can quantitative CT texture analysis be used to differentiate subtypes of renal cell carcinoma? Clin Radiol (2019) 74:287–94. 10.1016/j.crad.2018.11.009 30554807

[39] Limkin EJ Reuze S Carre A Sun R Schernberg A Alexis A The complexity of tumor shape, spiculatedness, correlates with tumor radiomic shape features. Sci Rep (2019) 9:4329. 10.1038/s41598-019-40437-5 30867443 PMC6416263

[40] Xu F Zhu W Shen Y Wang J Xu R Qutesh C Radiomic-Based Quantitative CT Analysis of Pure Ground-Glass Nodules to Predict the Invasiveness of Lung Adenocarcinoma. Front Oncol (2020) 10:872. 10.3389/fonc.2020.00872 32850301 PMC7432133

[41] Haas NB Manola J Uzzo RG Flaherty KT Wood CG Kane C Adjuvant sunitinib or sorafenib for high-risk, non-metastatic renal-cell carcinoma (ECOG-ACRIN E2805): a double-blind, placebo-controlled, randomised, phase 3 trial. Lancet (2016) 387:2008–16. 10.1016/S0140-6736(16)00559-6 PMC4878938 26969090

[42] Motzer RJ Haas NB Donskov F Gross-Goupil M Varlamov S Kopyltsov E Randomized Phase III Trial of Adjuvant Pazopanib Versus Placebo After Nephrectomy in Patients With Localized or Locally Advanced Renal Cell Carcinoma. J Clin Oncol (2017) 35:3916–23. 10.1200/JCO.2017.73.5324 PMC6018511 28902533

