# Supplementary material for: T1 Stage Clear Cell Renal Cell Carcinoma: A CT-Based Radiomics Nomogram to Estimate the Risk of Recurrence and Metastasis
Source: Front Oncol. 2020 Nov 4;10:579619. doi: 10.3389/fonc.2020.579619 (PMC7672185; doi:10.3389/fonc.2020.579619)
Supplement: Supplementary file 1 [file DataSheet_1.docx]

Supplementary Material

# Supplementary Information

## The Patient Recruitment Pathway

During the 7-year recruiting period, 508 consecutive patients with T1 stage ccRCC were operated on in our institution. Applying the inclusion and exclusion criteria, a total of 338 T1 stage ccRCC patients who underwent contrast enhanced CT prior to surgical resection were identified from our electronic hospital information system. 34 Patients with distant metastasis at the primary ccRCC diagnosis were defined as synchronous metastasis. For the rest of patients, chest-abdomen-pelvis CT examinations were performed every 6 months. 16 patients exhibit recurrence identified by imaging, or biopsy, and the median and maximum follow-up was 12 and 60 months, respectively. 118 patients without evidence of recurrence had a median and maximum follow-up of 41 and 86 months, respectively. 170 patients with a short-term follow-up, less than 3 years were excluded.

## CT Image Acquisition Parameters

The contrast enhanced CT scans were performed using a 320-detector CT scanner (Aquilion ONE, TOSHIBA) and a 64-detector CT scanner (Discovery, GE Healthcare). Table E1 showed the CT image acquisition details. Following unenhanced CT images, the corticomedullary phase (CMP, 30s) and nephrographic phase (NP, 90s) were obtained for all patients. Eighty milliliters of nonionic contrast agent (Omnipaque 350, GE Healthcare, Shanghai, China) was administered into an antecubital vein using a mechanical power injector (Ulrich CT Plus 150, Ulrich Medical, Ulm, Germany) at rate of 3 ml/s.

## Radiomic Feature Extraction Methodology

Texture variables included four categories of features and 396 quantitative variables comprising the characteristics of 10 Haralick, 42 histograms, nine form factor, and 60 gray level run length matrix (GLRLM) features with an offset of 1/4/7, and 48 gray level co-occurrence matrix (GLCM) features with an offset of 1/4/7.

# Supplementary Tables

**Supplementary Table 1. CT Image Acquisition Parameters**

| CT scanner | 320-detector CT scanner (Aquilion ONE) | 64-detector CT scanner (Discovery 750) |
| --- | --- | --- |
| Manufacturer | TOSHIBA | GE |
| Gantry rotation time (s) | 0.5 | 0.6 |
| Tube voltage (kV) | 120 | 120 |
| Tube current | automatic tube current | automatic tube current |
| Detector collimation (mm) | 80×0.5 | 64×0.625 |
| Matrix | 512×512 | 512×512 |
| Pitch | 1.388 | 0.984 |
| Slice thickness (mm) | 5 | 5 |

s, second; kV, kilovolt; mm, millimeter
