# Supplementary figures and images for: T1 Stage Clear Cell Renal Cell Carcinoma: A CT-Based Radiomics Nomogram to Estimate the Risk of Recurrence and Metastasis
Source: Front Oncol. 2020 Nov 4;10:579619. doi: 10.3389/fonc.2020.579619 (PMC7672185; doi:10.3389/fonc.2020.579619)

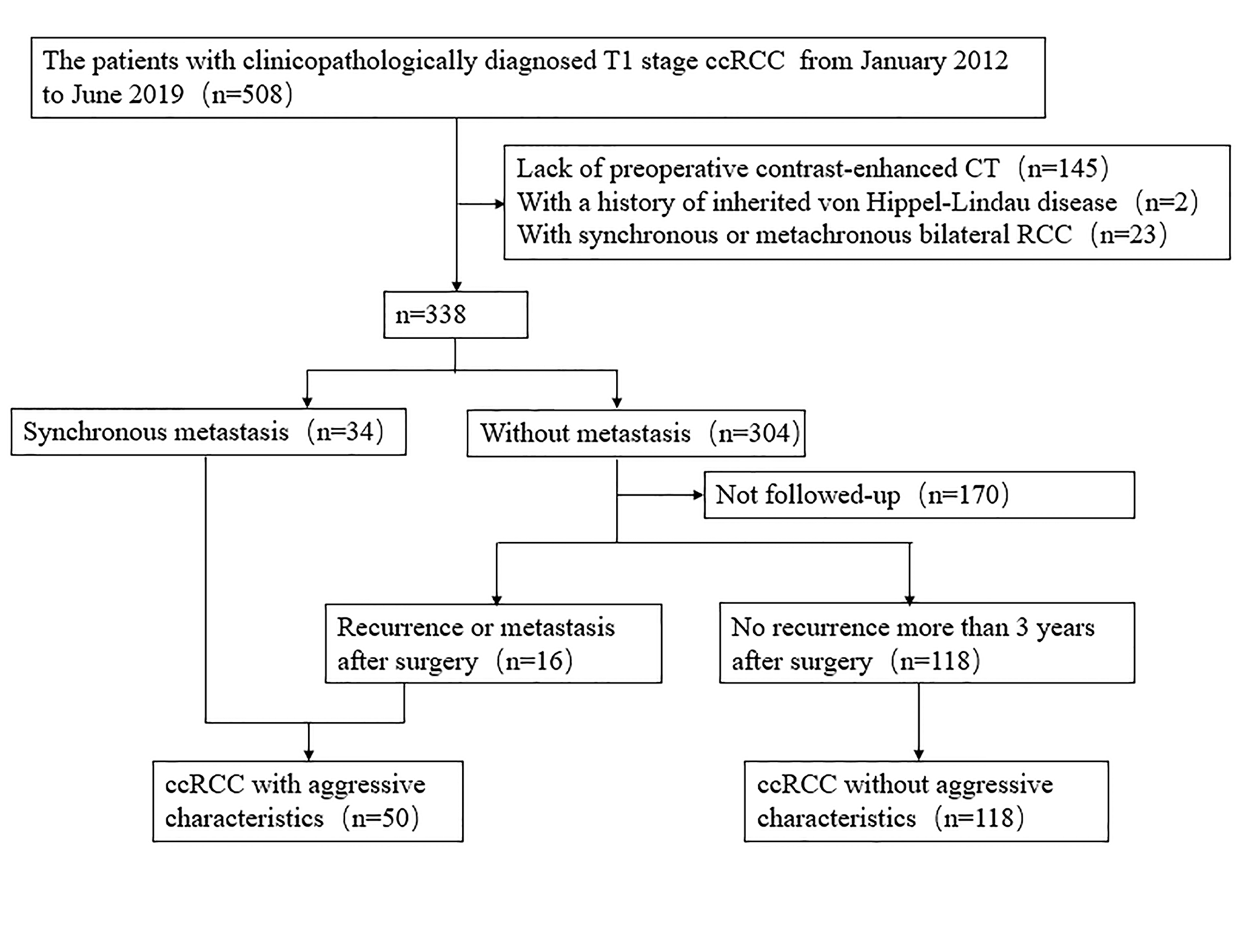

Supplement: Supplementary file 2 [file DataSheet_2.zip › FIG/Fig.1.tif]

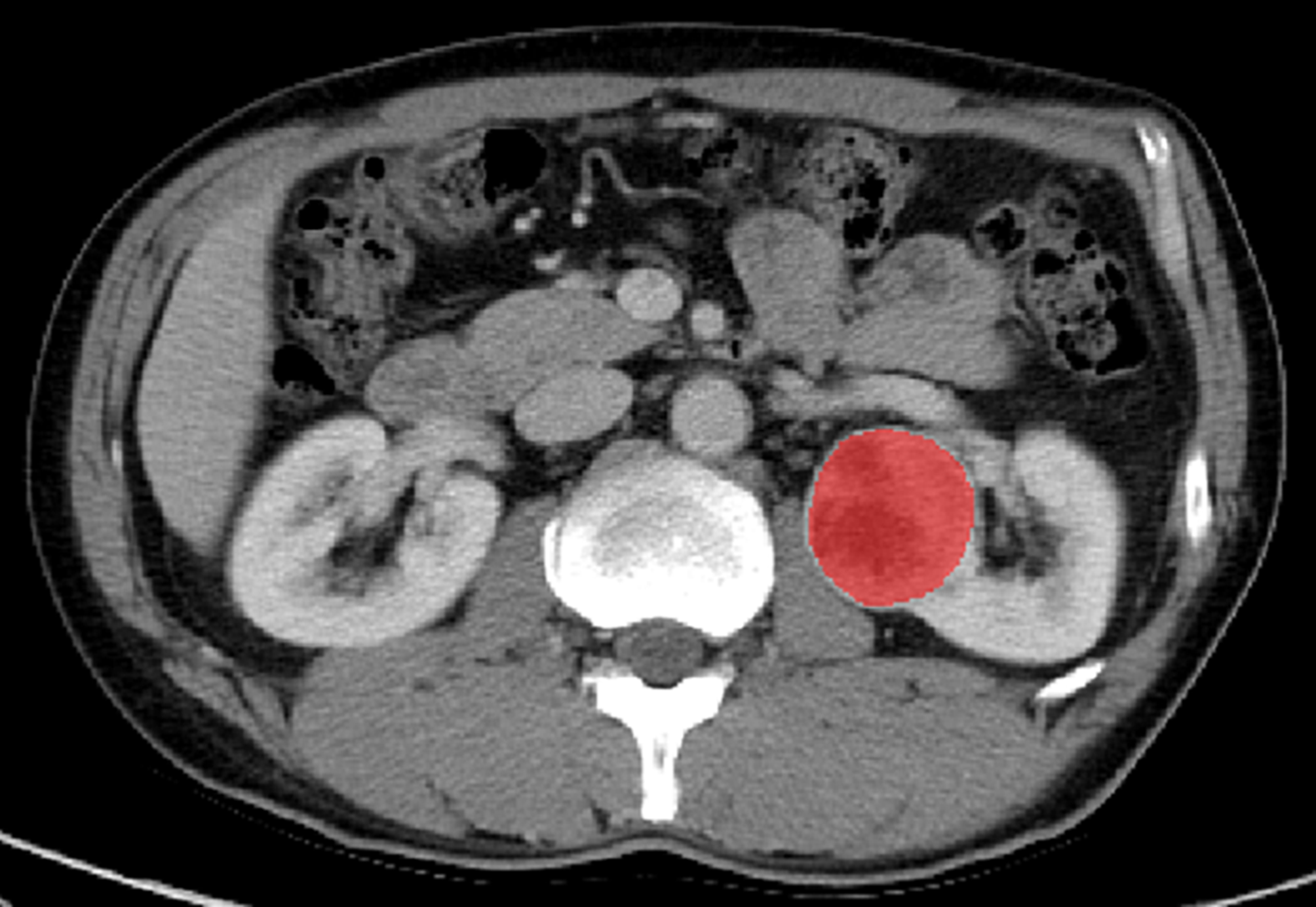

Supplement: Supplementary file 2 [file DataSheet_2.zip › FIG/Fig.2.tif]

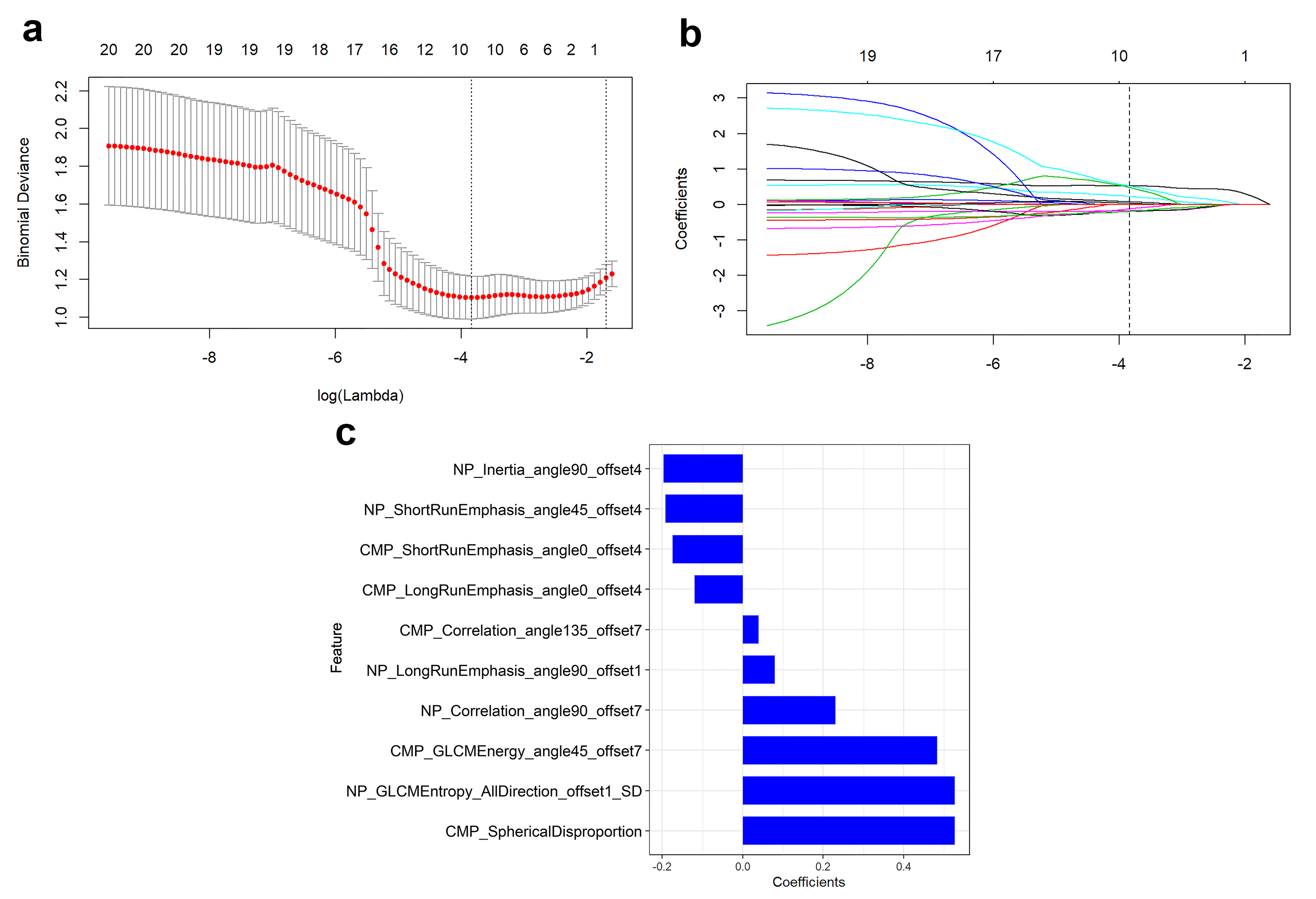

Supplement: Supplementary file 2 [file DataSheet_2.zip › FIG/Fig.3.tif]

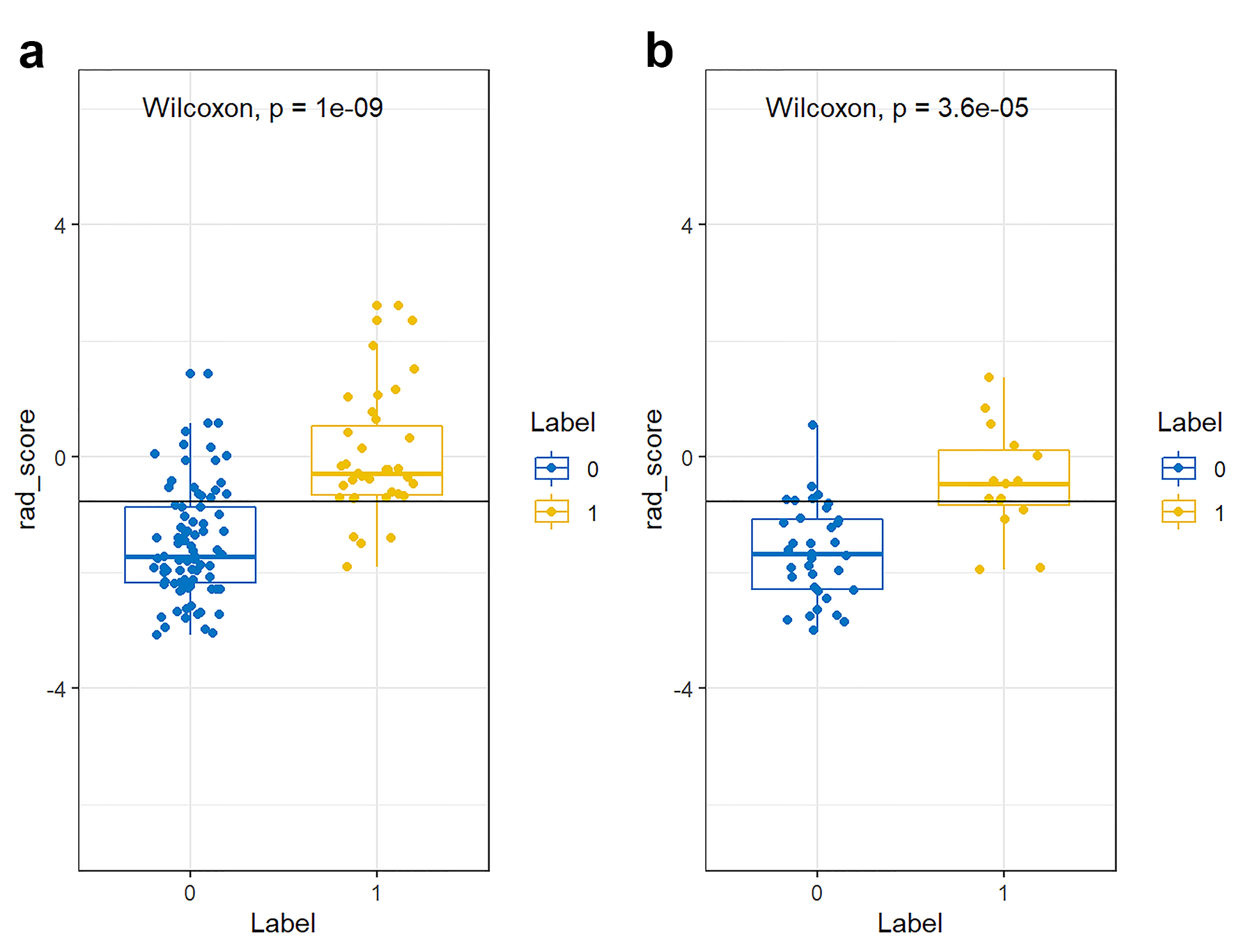

Supplement: Supplementary file 2 [file DataSheet_2.zip › FIG/Fig.4.tif]

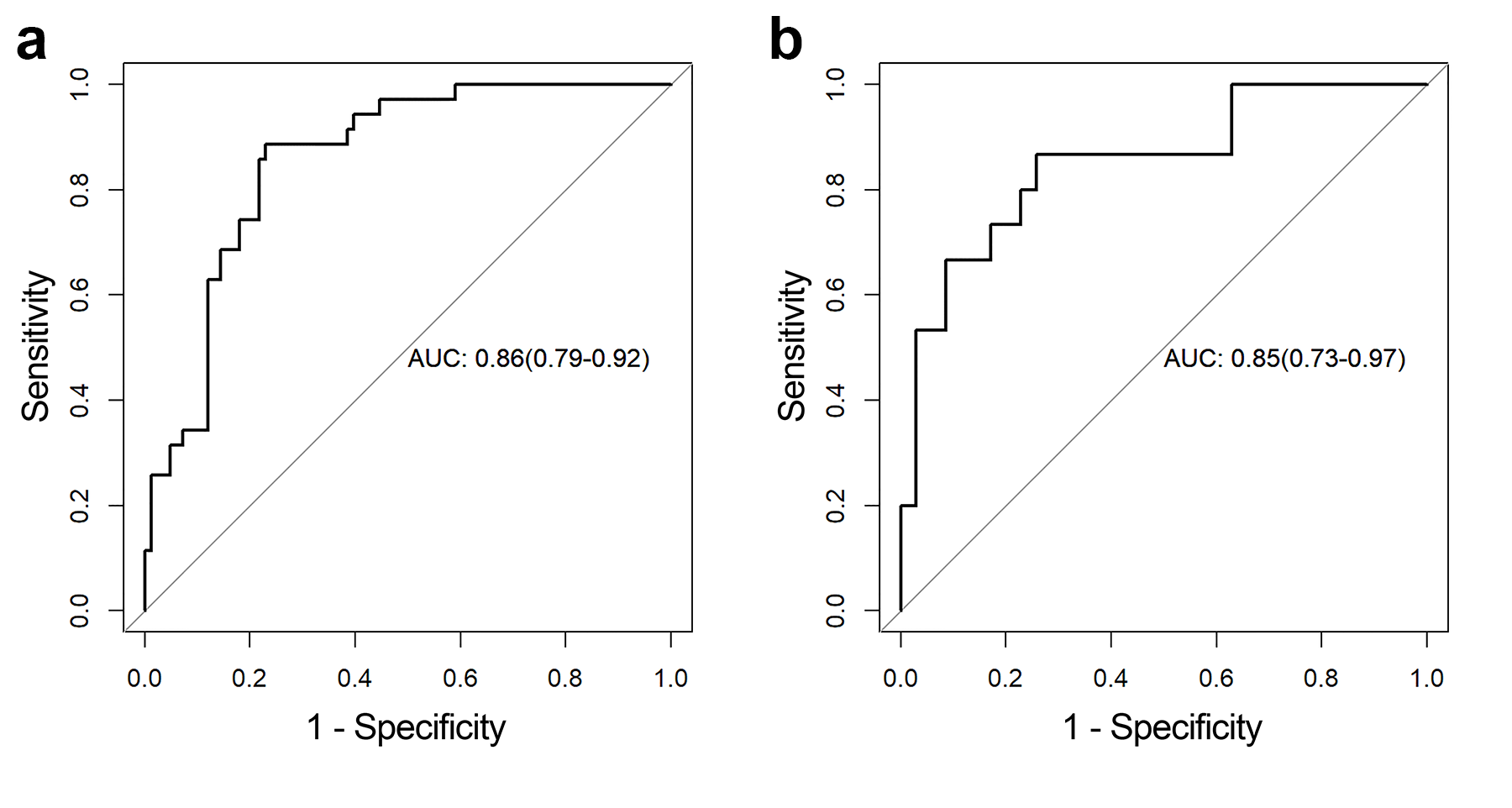

Supplement: Supplementary file 2 [file DataSheet_2.zip › FIG/Fig.5.tif]

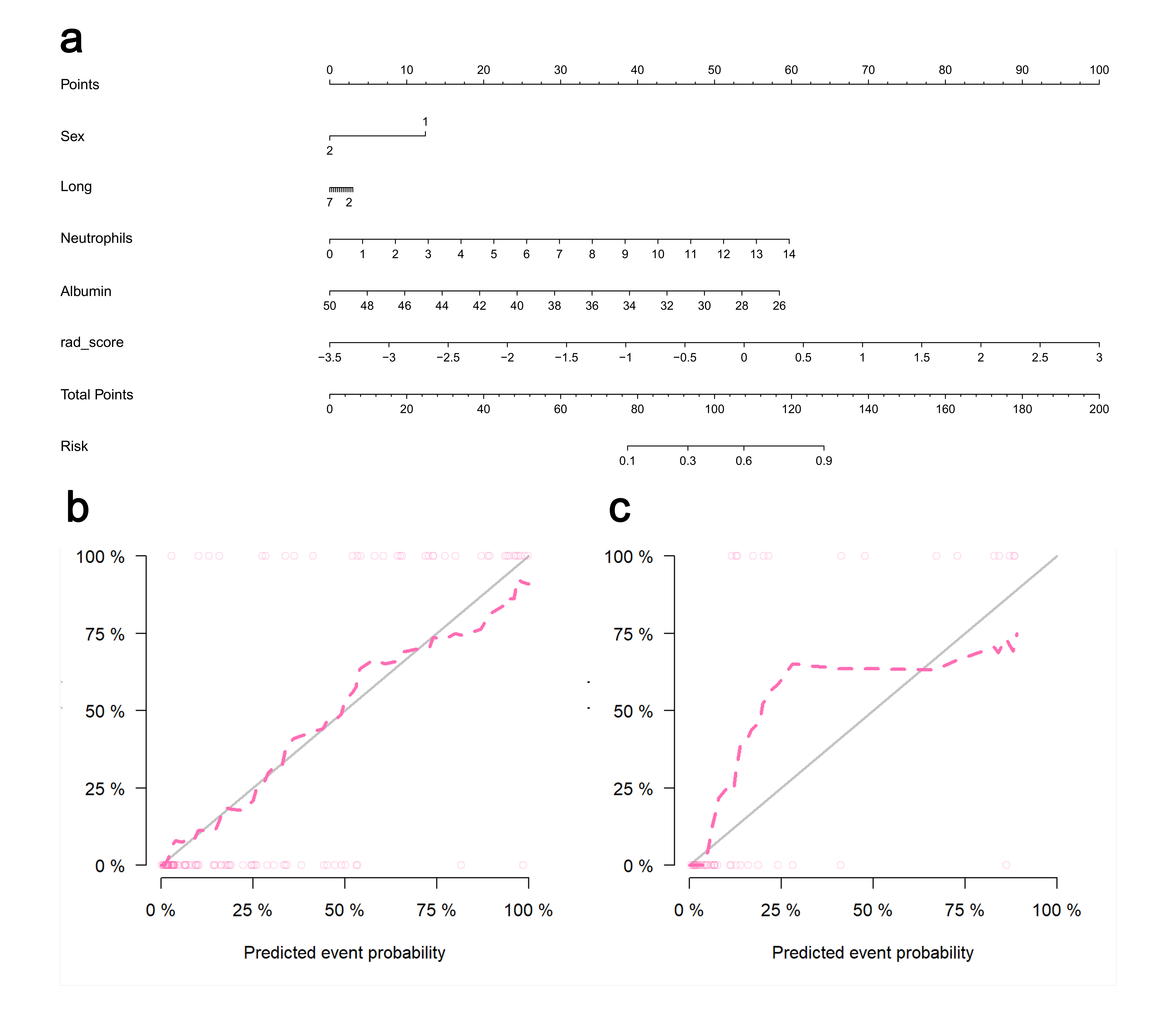

Supplement: Supplementary file 2 [file DataSheet_2.zip › FIG/Fig.6.tif]

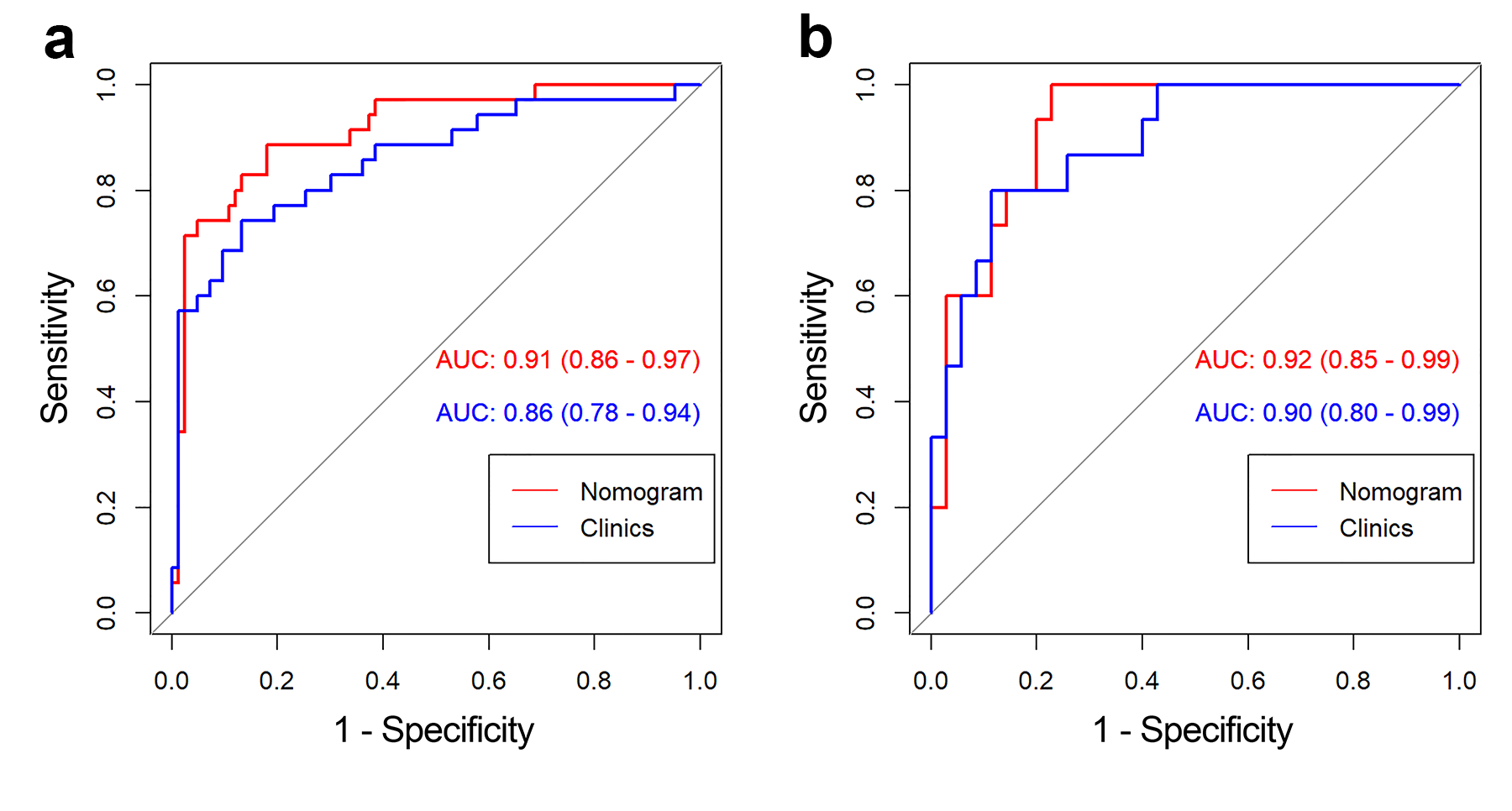

Supplement: Supplementary file 2 [file DataSheet_2.zip › FIG/Fig.7.tif]

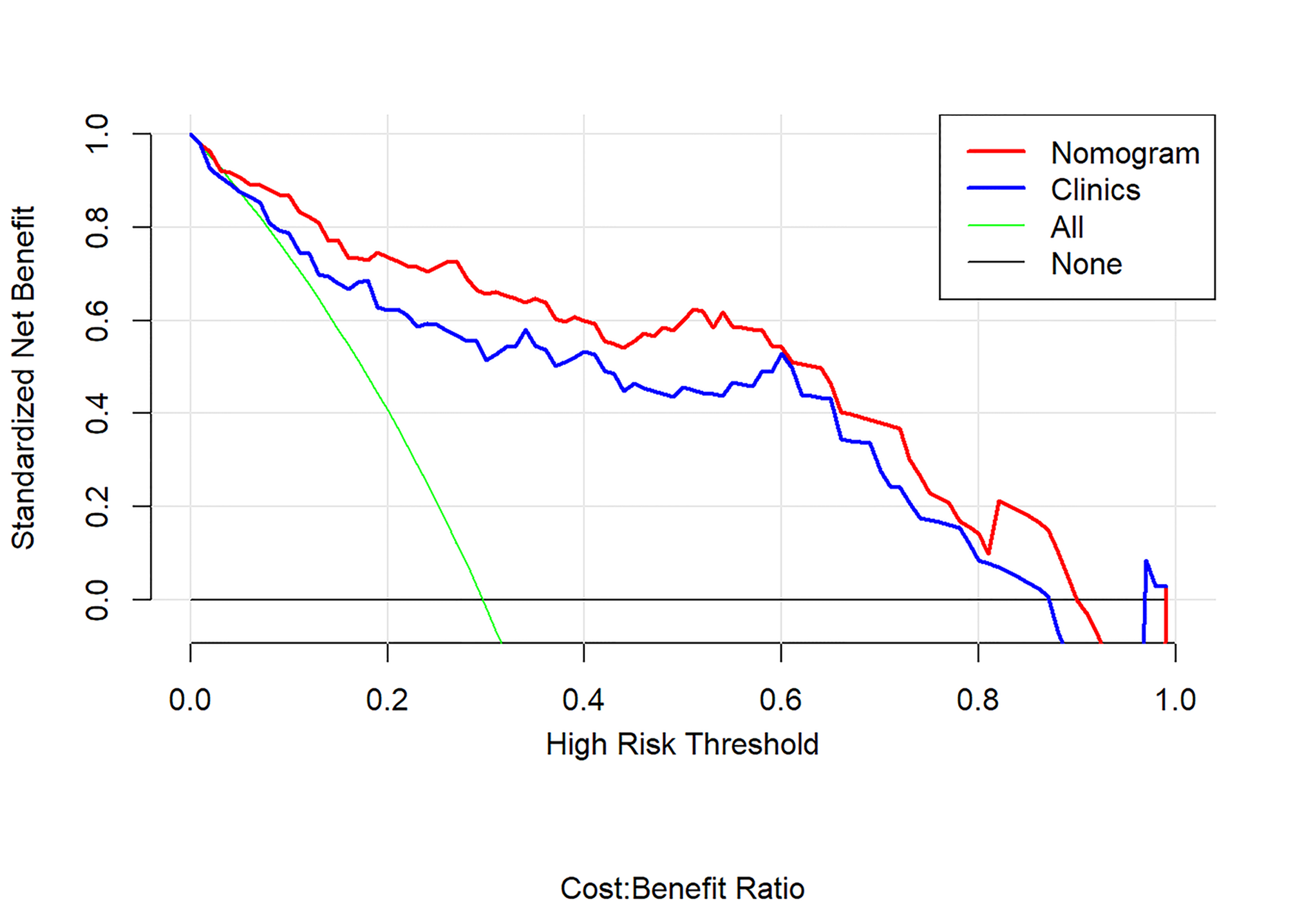

Supplement: Supplementary file 2 [file DataSheet_2.zip › FIG/Fig.8.tif]
